# Successful Aging: A Comprehensive Outcome Using Latent Profile Analysis

**DOI:** 10.1093/geroni/igaa057.1370

**Published:** 2020-12-16

**Authors:** Kasey Longley, Joseph Grzywacz

**Affiliations:** Florida State University, Tallahassee, Florida, United States

## Abstract

Understanding “successful aging” is a primary goal of gerontology and adult development researchers that has been motivated by rapid the increases in life expectancy over the last 100 years. Successful aging, as it is understood by Rowe and Kahn, is conceptualized multidimensionally in terms of limited disease and disability, high physical, mental and cognitive functioning, and active engagement with life. “Success” in all three domains reflects the idealized manifestation of “successful aging.” Nevertheless, research on successful aging typically focuses on only one or two aspects of the model – most commonly physical disease or disability. The overall goal of this research is to advance understanding and subsequent attempts to promote holistic successful aging. Specifically, using key metrics from each domain of successful aging from the Midlife Development in the United States (MIDUS), this study characterizes distinct profiles of successful aging, and it describes the distribution of these profiles in the adult population. Results indicate 3 profiles. These are labeled as Successfully Aged, Somewhat Successfully Aged, and Least Successfully Aged. Approximately 82.1% of the population (mean age=50.5) is classified as Successfully Aged, whereas the remainder are classified in the Somewhat Successfully Aged (12.2%), and Least Successfully Aged (5.6%), respectively. As expected, those who were classified as Successfully Aged had the highest cognitive scores, sense of well-being, and self-rated health; and had the lowest number of age-related physical disabilities (i.e. cancer, stroke, osteoporosis, etc.) and mental health conditions (depression, anxiety disorder, and panic disorder). This outcome can be applied to multiple predictors.

